# On the Prevention of Eye Accidents Occurring in Trades

**Published:** 1900-11

**Authors:** 


On the Prevention of Eye Accidents Occurring in Trades. By Simeon Snell, F. R. C. S. Edin., Ophthalmic Surgeon to the Royal Infirmary, and Professor of Ophthalmology in the University College, Sheffield. London: Bale, Sons & Danielsson, Ltd., 83–89 Oxford street, W. [Price, 1 shilling.]

This reprint from the *Brit. Med. Jour.* of Dr. Snell's presidential address before the section of ophthalmology of the British Medical Association, 1899, is a very tastily gotten up little book with parchment covers and uncut leaves. The subject matter is of interest to every ophthalmologist and general practitioner who sees so many cases of this sort. In calling attention to the constant menace to the eyesight many occupations involve, the author has performed a timely service and it is unfortunate that we do not have the subject more frequently discussed. The author describes his wire mesh eye protectors to be worn for various occupations. This is no better and probably would prove more tiring to the eyes than the mica eye protectors, worn to some extent in this country. This latter he does not mention.

The illustrations showing the implements used by workmen to remove foreign bodies and some of the occupations most dangerous to sight are interesting. R. H. S.

